# The circACTN4 interacts with FUBP1 to promote tumorigenesis and progression of breast cancer by regulating the expression of proto-oncogene MYC

**DOI:** 10.1186/s12943-021-01383-x

**Published:** 2021-06-11

**Authors:** Xiaosong Wang, Lei Xing, Rui Yang, Hang Chen, Min Wang, Rong Jiang, Luyu Zhang, Junxia Chen

**Affiliations:** 1 Department of Cell Biology and Genetics, Chongqing Medical University, #1 Yixueyuan Road, Chongqing, 400016 China; 2 Department of Endocrine and breast surgery, The First Affiliated Hospital of Chongqing Medical University, #1 Yixueyuan Road, Chongqing, 400016 China; 3 Laboratory of Stem Cells and Tissue Engineering, Chongqing Medical University, #1 Yixueyuan Road, Chongqing, 400016 China; 4 Molecular Medicine and Cancer Research Center, Chongqing Medical University, #1 Yixueyuan Road, Chongqing, 400016 China

**Keywords:** circactn4, FUBP1, The RNA-binding protein, MYC, Breast cancer

## Abstract

**Background:**

Recent studies have revealed that circular RNAs (circRNAs) play significant roles in the occurrence and development of many kinds of cancers including breast cancer (BC). However, the potential functions of most circRNAs and the molecular mechanisms underlying progression of BC remain elusive.

**Method:**

Here, Circular RNA microarray was executed in 4 pairs of breast cancer tissues and para-cancer tissues. The expression and prognostic significance of circACTN4 in BC cells and tissues were determined by qRT-PCR and in situ hybridization. Gain-and loss-of-function experiments were implemented to observe the impacts of circACTN4 on the growth, invasion, and metastasis of BC cells in vitro and in vivo. Mechanistically, chromatin immunoprecipitation, luciferase reporter, RNA pulldown, mass spectrum, RNA immunoprecipitation, fluorescence in situ hybridization and co-immunoprecipitation assays were executed.

**Results:**

CircACTN4 was significantly upregulated in breast cancer tissues and cells, its expression was correlated with clinical stage and poor prognosis of patients with BC. Ectopic expression of circACTN4 strikingly facilitated the growth, invasion, and metastasis of breast cancer cells in vitro and in vivo. Whereas knockdown of circACTN4 revealed opposite roles. CircACTN4 was mainly distributed in the nucleus. Further mechanistic research proved that circACTN4 could competitively bind to far upstream element binding protein 1 (FUBP1) to prevent the combination between FUBP1 and FIR, thereby activating MYC transcription and facilitating tumor progression of breast cancer. Furthermore, we found that upstream transcription factor 2 (USF2) might promote the biogenesis of circACTN4.

**Conclusion:**

Our findings uncover a pivotal mechanism that circACTN4 mediated by USF2 might interact with FUBP1 to promote the occurrence and development of breast cancer via enhancing the expression of MYC. CircACTN4 could be a novel potential target for diagnosis and treatment of breast cancer.

**Supplementary Information:**

The online version contains supplementary material available at 10.1186/s12943-021-01383-x.

## Background

Breast cancer is the malignant tumor with the highest incidence among women in the world. Globally, about 2.1 million cases of breast cancer were newly diagnosed in 2018, accounting for about 25% of all female malignant tumor cases. Although the early detection and effective systematic treatment of breast cancer have improved, it remains the leading cause of cancer death in women all over the world [1]. Thus, there is an urgent need to find new therapeutic targets and strategies for BC.

Circular RNAs (circRNAs) are a new class of RNAs that have been widely found in various species through high-throughput sequencing in recent years. CircRNAs are covalently closed, single-stranded transcripts generated by pre-mRNA back-splicing without 5′ caps and 3′ tails. Due to the covalent loop structure, circRNAs are resistant to RNA exonucleases and are more stable than linear RNA [2]. CircRNAs reveal disease-specific and development stage-specific features in different pathological circumstances, which suggests that circRNAs could be served as novel potential biomarkers for diagnosis and treatment [3]. Recent researches have demonstrated that circRNAs have important potential roles in regulating gene expression. CircRNAs exert their functions, including as miRNA sponges, RNA-binding protein scaffolds, intermediates in RNA alternative splicing (AS), protein translation templates and transcription regulators [4]. However, the interaction of circRNAs and proteins were less studied in human cancers.

Accumulating evidence has indicated that the dysregulated circRNAs are involved in the development of various cancers. The most commonly reported mechanism of circRNAs can function as miRNA sponges to relieve the suppression for miRNA targeted gene expression [5–7]. In breast cancer, circRNAs, such as circRNA_0006528, circRAD18, circHMCU and circRNF20, have been revealed powerful potential in regulating cell proliferation, migration, metabolism and tumor progression, and their expressions are correlated with high TNM stage and prognosis [8–11]. Recently, researches have reported that circRNAs could interact with RNA binding proteins (RBPs) to exert biological functions [12, 13]. For example, circular RNA CDR1as could directly combine with the p53 DBD domain, thus disrupting the p53/MDM2 complex formation to suppress gliomagenesis [14]. Circ0005276 increases the proliferation and mobility of prostate cancer cells through binding with FUS to transcriptionally activate XIAP [15]. The RNA-binding protein RBM3 regulates the generation of circular RNA SCD-circRNA 2 to facilitate cell proliferation in hepatocellular carcinoma [16]. However, the interaction and relationship between circRNAs and proteins in breast cancer remain largely unknown.

The human far upstream element (FUSE) binding protein 1 (FUBP1), a multifunctional DNA- and RNA-binding protein, is implicated in transcription, translation, and RNA splicing cellular processes. It is well known that FUBP1 can bind to FUSE upstream of c-MYC promoter to recruit and activate transcription factor TFIIH, thus promoting the transcription of c-myc gene. FUBP1 also might bind with the FBP interacting repressor (FIR) to form inhibitory complexes with FUSE and TFIIH, and inhibit c-MYC transcription. The interplay of FUSE, FUBP1 and FIR in this molecular machinery can fine-tune c-myc transcription in a real-time manner [17]. FUBP1 overexpression was found in many cancers and led to a dysregulation of targets including MYC oncogene [18]. The study have shown that the expressions of FUBP1 and its target MYC in breast cancer tissues were higher than those in adjacent normal tissues [19]. It has been reported that lncRNA SNHG1 could directly interact with FUBP1 central domain and resist the binding between FIR and FUBP1, thereby modulating the oncogene MYC transcription and promoting cancer development [20]. To date, the association of between circRNAs and FUBP1 has not been explored.

In this study, we performed circular RNA microarray and displayed the circRNA expression profile in breast cancer tissues. Then we focused on a novel circRNA circACTN4 (hsa_circ_0050900) from ACTN4 gene and investigated its biological functions as well as the underlying molecular mechanisms in the breast cancer development. The results showed that circACTN4 was highly expressed in BC tissues and cells, which was positively correlated with advanced tumor stage and poor prognosis. Moreover, we demonstrated that USF2 could facilitate the generation of circACTN4. Our functional investigation displayed that circACTN4 could enhance BC cell growth and metastasis. Further mechanistic research revealed that circACTN4 could competitively bind to FUBP1 and block the binding of FUBP1 with FIR, thus promoting the transcription of MYC and development of breast cancer. Our results suggest that circACTN4 could be a new therapeutic target and promising prognostic biomarker for BC.

## Materials and methods

### Clinical BC tissues and cell lines

Cancer and para-cancer tissues of 80 breast cancer patients who did not receive preoperative chemotherapy or radiotherapy were obtained at the First Affiliated Hospital of Chongqing Medical University (Chongqing, China). Fresh tissues were saved in liquid nitrogen or stored at − 80 °C until RNA extraction. All procedures were implemented according to the guidelines of the Declaration of Helsinki. All patients provided written informed consent. Human normal breast epithelial MCF-10A cells and breast cancer MCF-7 and SK-BR-3 cells as well as 293 T cells were preserved in our laboratory, breast cancer ZR-75-1, BT-474 and T-47D cells were bought from American Type Culture Collection (ATCC, Manassas, VA, USA). MCF-10A cells were cultivated in MEBM BulletKit medium (Lonza, Basel, Switzerland), MCF-7, T-47D and 293 T cells were maintained in DMEM (Gibco, Carlsbad, CA, USA) with 10% fetal bovine serum (FBS), SK-BR-3, ZR-75-1 and BT-474 were fostered in RPMI-1640 (Gibco, Carlsbad, CA, USA) with 10% or 20% FBS. All media were supplemented with 1% penicillin/streptomycin. All cells were placed in a 5% CO_2_ incubator at 37 °C.

### Plasmid construction, RNAi and stable transfection

circACTN4 overexpression vector were designed and the human full-length circACTN4 was inserted into vector pLC5-ciR by Geenseed (Guangzhou, China), the mock vector without circACTN4 sequence was served as a control. To knock down circACTN4, siRNAs (small interfering RNAs) targeting back splice junction of circACTN4 (si-circ#1, si-circ#2) and siRNA-NC were synthesized by Geenseed (Guangzhou, China). Human circACTN4 gene was inserted into the lentivirus vector CV146 (Ubi-MCS-SV40-firefly_Luciferase-IRES-Puromycin). The si-circ#2 was subcloned into the lentivirus vector GV344 (hU6- MCS- Ubiquitin- firefly_Luciferase- IRES- puromycin) by Genechem (Shanghai, China). Overexpression vectors of human USF2, FUBP1, FIR and ACTN4 gene were synthesized and ligated into GV146 (CMV-MCS-IRES-EGFP-SV40-Neomycin) by Genechem (Shanghai, China). Short-hairpin RNAs (shRNAs) directed against USF2, FUBP1, FIR and ACTN4 gene were constructed in GV102 (hU6-MCS-CMV-GFP-SV40-Neomycin) vectors (Genechem Co., Ltd., Shanghai, China). The lentiviral vector was transiently transfected into HEK293T cells. The viral supernatant was collected for animal experiments. Stable cell lines were screened with neomycin or puromycin. All transfections were implemented using Lipofectamine 3000 (Invitrogen, Carlsbad, CA, USA) according to manufacturer’s instructions. The sequences of shRNAs and siRNAs used were displayed in Additional file 1: Table S2.

### Animal experiments

Four-week-old female BALB/c nude mice were injected subcutaneously with 10^7^ MCF-7 cells infected with various lentiviruses. All processes were executed according to NIH Guidelines for the Care and Use of Laboratory Animals and approved by Chongqing Medical University Animal Care and Use Committee. The width and length of the tumor were measured once every week, and tumor volume was calculated based on the formula (length × width^2^/2). Mice were sacrificed and tumors were harvested and weighed after 4 weeks. The tumors and lungs were obtained for the next study. The mice were administered subcutaneously with cells infected with the lentiviral vector overexpressing circACTN4 or the mock lentiviral respectively (*n* = 10 per group) for survival analysis, and were observed for 2 months as a cutoff. After 2 months, the mice still alive were regarded to be censored, the mice were executed and the livers were excised for pathological analysis. MCF-7 cells were stably infected with luciferase-labeled mock, overexpressed circACTN4, small interfering control (si-NC), and si-circ#2 lentivirus. 4 × 10^6^ cells were injected into female BALB/c nude mice tail vein. After 4 weeks, 150 mg/kg D-Luciferin (Sciencelight Biology Science & Technology Co., Ltd. Shanghai, China) was injected by intraperitoneal injection, After 15 min, the mice were imaged for luciferase activity using the Nightowl LB 983 in vivo imaging system (Berthold, Germany).

### Microarray analysis

Total RNA of 4 pairs of non-triple negative breast cancer and para-cancer tissues was extracted with TRIzol reagent (Takara, Dalian, China) and quantified by the NanoDrop ND-1000 (Thermo Fisher Scientific, USA), then digested with RNase R to eliminate linear RNAs and enrich circular RNAs. The sample preparing and microarray hybriding were executed following the Arraystar’s standard protocols by KangChen Bio-tech (Shanghai, China). Microarray analysis of circRNA was implemented by Arraystar Human circRNA Array V2. preparing sample.

### Nuclear-cytoplasmic fractionation, RT-PCR and qRT-PCR assays

Total RNA from tissues and cells was extracted with TRIzol reagent (Takara, Dalian, China). Nuclear and cytoplasmic RNA in MCF-7 cells was isolated using the PARIS™ Kit (Life Technologies, Austin, Texas, USA) according to the manufacturer’s protocols. RNase R (Epicentre Biotechnologies, Madison, WI, USA) digestions with 4 U/μg were performed for 0, 10, 20, 30 and 40 min at 37 °C. One hundred nanograms per milliliter actinomycin D (Cell Signaling Technology, Beverly, MA, USA) was used to treat total RNA from BC cells against new RNA synthesis for 12 and 24 h. The semi-quantitative PCR amplification cycle was 39. Reverse transcription was conducted by PrimeScript RT Reagent Kit (Takara, Dalian, China), and quantitative real-time PCR (qRT-PCR) analysis was executed with the TB Green Premix Ex Taq (Takara, Dalian, China). The 2^-ΔΔCt^ method was used to analyze the relative expression levels. The primer sequences were listed in Additional file 1: Table S1.

### Fluorescence in Situ Hybridization (FISH) and Immunofluorescence (IF)

Cy3-labeled probe for circACTN4 was synthesized (Geneseed, Guangzhou, China). BC cells were cultured on coverslips, incubated with antibodies specific for FUBP1 (Thermo fisher scientific, USA) at 4 °C overnight, and treated with FITC-labeled goat anti-rabbit IgG (Bosterbio, Wuhan, China; 1:1000 dilution) at 37 °C for 2 h, and then incubated with FISH probe in hybridization buffer (Geneseed, Guangzhou, China) at 37 °C for 16 h. the cell nuclei were stained by DAPI(4′6- diamidino- 2- phenylindole). These images were taken with Olympus BX51 fluorescence microscope (Tokyo, Japan). The probe sequences were displayed in Additional file 1: Table S3

### Tissue Microarray (TMA) and In Situ Hybridization (ISH)

TMA was produced from 240 paraffin-embedded samples by Outdo Biotech (Shanghai, China). ISH was used to detect the expression of circACTN4 in BC tissues. First, the TMA was dewaxed in xylene and rehydrated with 100, 95, 85 and 75% alcohol, and then the tissues were hybridized with specific digoxin-labeled circACTN4 probe (Geneseed, Guangzhou, China). The specimens were incubated with AP-conjugated anti-digoxin antibody (Roche, Basel, Switzerland) at 4 °C overnight, than stained with NBT/BCIP (Roche, Basel, Switzerland) and photographed. The expressions of circACTN4 were quantified and analyzed. The positive staining intensity (0, negative; 1, weak; 2, moderate; 3, strong) was multiplied by the percentage of positive staining cells (0, < 10% = 0; 1, 10–25%; 2, 26–50%; 3, 51–75%; 4, > 75%) to calculate the score for ISH staining. The ISH score ≤ 8 defined low expression, while >8 indicated high expression. The probe sequence was displayed in Additional file 1: Table S3.

### Dual luciferase reporter assay

The human ACTN4 promoter luciferase reporter gene vector was constructed, the full-length promoter of ACTN4 carrying mutant or wild type was respectively cloned into pGL3-basic vectors (GeneCreate, Wuhan, China), and co-transfected with USF2 overexpression vector or mock vector into 293 T cells. The cells were harvested after 48 h, the Dual Luciferase Reporter Assay System Kit (Promega, Madison, WI, USA) was used to detect the activities of firefly and Renilla luciferase.

### Cell proliferation, wound healing, migration and invasion assays

CCK-8, Edu, colony formation, wound healing, transwell migration and invasion assays were carried out as previously reported [21].

### Cell cycle and apoptosis analysis

Forty-eight hours after transfection, cells were resuspended and fixed with ice cold 70% ethanol overnight at 4 °Cfor cell cycle analysis, and stained by PI (propidium iodide), than tested using the flow cytometry (Becon Dickinson FACS Calibur, NY, USA). The cells were stained with Annexin V-FITC and PI to detect the cell apoptosis rate with flow cytometry. Hoechst 33342 (Beyotime, Shanghai, China) staining was also utilized to detect cell apoptosis following the manufacturer’s instructions and photographed with the fluorescent microscope (Leica, Wetzlar, Germany).

### Chromatin immunoprecipitation (ChIP) assay

ChIP assay was undertaken using EZ ChIP Chromatin Immunoprecipitation Kit (Millipore, Billerica, MD, USA) following the manufacturer’s protocols. Briefly, 2 × 10^7^ cells were used for each individual reaction. The MCF-7 cells were fixed by 1% formaldehyde and lysed. The genome was sonicated 200 ~ 1000 bp DNA fragments. Ten microgram antibodies specific for USF2 (Abcam, Burlingame, CA, USA), FUBP1 (Thermo Fisher Scientific, USA) and FIR (Cell Signaling Technology, Beverly, MA, USA) were added into the cell lysate for incubation at 4 °C overnight, than incubated with Protein A Agarose/Salmon Sperm DNA beads (50% Slurry). The input DNA and immunoprecipitated DNA was purified and detected by qPCR using the primer sequences (Additional file 1: Table S1).

### RNA pull down assay and mass spectrometry

The interaction between circACTN4 and RNA-binding-protein was detected by Pierce Magnetic RNA-Protein Pull-Down Kit (Thermo Fisher Scientific, USA) according to the manufacturer’s protocols. Biotin-labeled probes targeting junction site of circACTN4 were synthesized by RiboBio (Guangzhou, China) and oligo probe was used as a control. Linear circACTN4 was transcribed with Biotin RNA Labeling Mix (Roche) and T7 RNA polymerase (Thermo Fisher Scientific, USA), circularized using T4 RNA ligase I and digested by RNase R. The cells were lysed and incubated with biotin-labeled circACTN4 probe. Afterwards, cell lysates were subjected to streptavidin agarose magnetic beads at normal temperature. Finally, interacting proteins were identified by mass spectrometry and Western blot (Life Sciences Institute of Chonqing Medical University, Chongqing, China). The sequences of the probe were shown in Additional file 1: Table S3.

### RNA immunoprecipitation (RIP) assay

The interaction between RBP FUBP1 and circACTN4 was detected by RNA immunoprecipitation kit (Geneseed, Guangzhou, China) according to the manufacturer’s instruction. Briefly, 2 × 10^7^ cells were harvested and lysed by RIP lysis buffer, then incubated with magnetic beads conjugated with antibody against IgG (Cell Signaling Technology, Beverly, MA, USA) or FUBP1 (Thermo Fisher Scientific, USA). The coprecipitated RNAs were examined using qRT-PCR or RT-PCR with specific primers. The primer sequences were listed in Additional file 1: Table S1.

### Co-immunoprecipitation (co-IP) assay

MCF-7 cells were transfected with overexpressed circACTN4 plasmids and si-circ#2 by lipofectamine 2000 (Thermo Fisher Scientific, USA). Co-IP assay was carried out using a Co- Immunoprecipitation Kit (Millipore, Billerica, MD, USA) following the manufacturer’s protocols. In brief, cells were harvested and lysed, then incubated with 10μg IgG or specific for FUBP1 antibody (Thermo Fisher Scientific, USA) coated beads and rotated at 4 °C overnight. Bead-bound proteins were released and analyzed by Western blot.

### Immunohistochemistry (IHC)

For IHC assay, tissues were fixed with 4% paraformaldehyde and embedded in paraffin, then cutted into slices. Primary antibodies against MYC (1:8000, Proteintech,USA), CDK4 (1:8000, Proteintech, USA), CCND2 (1:200, Cell Signaling Technology, Beverly, MA, USA) and Ki-67 (1:8000, Proteintech,USA) were used. HRP-labeled streptavidin solution was added into the samples for 15 min after both primary and secondary antibody incubations. The immunocomplex was visualized with DAB, and the nucleus were counterstained with haematoxylin. Pictures were taken with a microscope (Leica, Wetzlar, Germany).

### Western blotting

Tissue and cellular proteins were extracted with radioimmunoprecipitation assay buffer (Beyotime, Jiangsu, China) mixed with phenyl-methylsulfonyl fluoride (PMSF), and quantified with BCA protein assay kit (Beyotime, Jiangsu, China). Equal amount of proteins were loaded and analyzed by 10% SDS-PAGE gels. The isolated proteins were transferred to PVDF membranes (Bio-Rad, CA, USA). After blocking with 5% fat-free milk, the membranes were incubated with primary antibodies against MYC (1:1000), FUBP1 (1:1000), FIR (1:1000), nm23-H1(1:1000), MMP2 (1:1000), MMP9 (1:1000), CDK4 (1:1000), CCND2 (1:1000), CCND1 (1:1000), CCNE1 (1:1000), Bcl-2 (1:1000), Bax (1:1000) and cleaved Caspase-3 (1:1000) (Cell Signaling Technology, Beverly, MA, USA) overnight at 4 °C. The membranes were washed by TBST, and then incubated with goat anti-rabbit secondary antibodies (Thermo Fisher Scientific, USA) for 2 h. Finally, the bands were illuminated with Pierce ECL (Thermo Fisher Scientific, USA).

### Statistical analysis

Statistical analyses were conducted by GraphPad Prism 7.0 (GraphPad Software Inc., CA, USA) and SPSS 21.0 (IBM, SPSS, Chicago, IL, USA). Data are expressed as the mean ± S.D. (Standard Deviation). The comparisons of two groups were computed using two-tailed Student’s t test, ANOVA or χ^2^ test. Correlations were analyzed by Pearson’s correlation test. Univariate and multivariate Cox proportional hazards regression models were used to identify factors associated with survival. The survival analysis was conducted with the Kaplan–Meier plots and the log-rank test. Differences were statistically significant at *P* < 0.05.

## Results

### CircACTN4 is identified and USF2 promotes expression of circACTN4 in BC

**Fig. 1:**
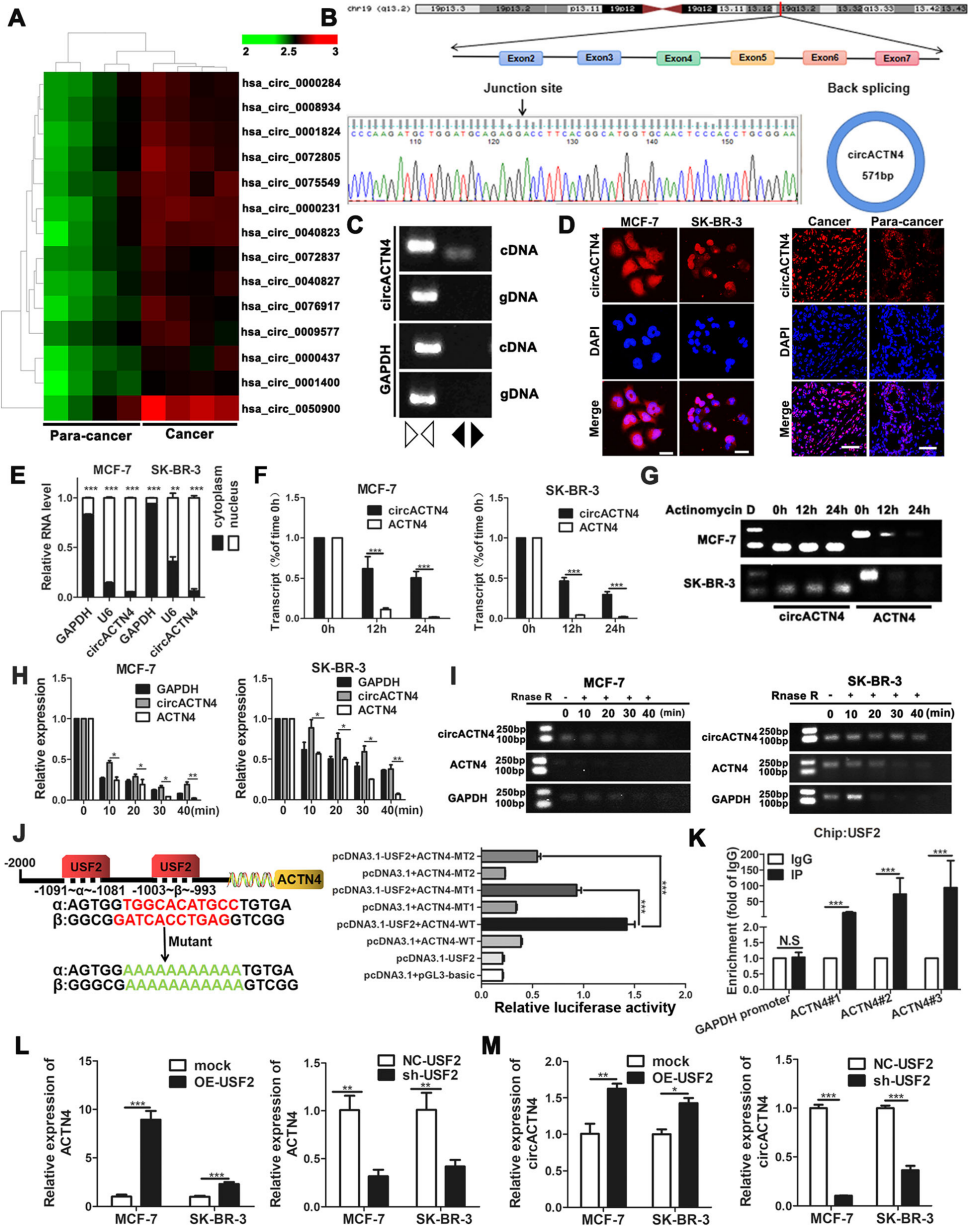
circACTN4 is verified and characterized in BC cells. **a** Heatmap showed 14 representative differentially upregulated circRNAs in BC tissues compared with adjacent normal tissues. The red and green represent the upregulated and downregulated circRNAs. **b** Schematic illustration of circACTN4 formation via the circularization from exons 2 to exon 7 in ACTN4 gene. The back-splice junction sequences were confirmed by Sanger sequencing. **c** The existence of circACTN4 from cDNA and gDNA in MCF-7 cell was detected by RT-PCR with the divergent and convergent primers. CircACTN4 could only be amplified by divergent primers from cDNA but not gDNA. **d** The localization of circACTN4 was investigated in BC cells (magnification,× 1000, Scale bar, 25um) and tissues (magnification,× 400, Scale bar, 50um) with FISH. **e** Nuclear-cytoplasmic fractionation assay displayed that circACTN4 was mostly distributed in the nucleus of BC cells. U6 was considered as a nuclear control and GAPDH was used as a cytoplasmic protein control. **f** and **g** The expressions of circACTN4 and ACTN4 of BC cells were analyzed by qRT-PCR and RT-PCR after treatment with actinomycin D. **h** The levels of circACTN4 and ACTN4 were detected after RNase R digestion at different time points by qRT-PCR. **i** RT-PCR was used to detect the expression of circACTN4 and ACTN4 after with RNase R treatment. **j** Schematic model of wild type (WT) and mutant (Mut) sequences of two putative binding sites of USF2 on ACTN4 promoter. The relative luciferase activities were detected in MCF-7 cells co-transfected with luciferase reporter plasmids containing wild type or mutant ACTN4 promoter sequence and overexpression plasmids of USF2. **k** ChIP-qPCR analysis showed that USF2 was enriched at the promoter region of ACTN4, and GAPDH promoter was used as negatively control. **l** and **m** The expression levels of circACTN4 and ACTN4 were determined in BC cells after USF2 up-regulation or down-regulation by qRT-PCR. GAPDH was used as the normalizing gene in the above experiments. The data are presented as the mean ± SD, *P < 0.05, **P < 0.01, ***P < 0.001

To investigate the function of circRNAs in the development of BC, the circRNA expression signatures in BC tissues were probed utilizing microarray analysis of 4 pairs of BC tissues and para-cancer tissues. When we use fold-change ≥2 and *P*-value < 0.05 as the cut-off criteria, we found 85 significantly differentially expressed circRNAs, including 63 up-regulated and 22 down-regulated circRNAs. The heatmap shows 14 upregulated circRNAs, among which circACTN4 (hsa_circ_0050900) was the most dysregulated one, and its expression in breast cancer tissues was 10 fold higher than that in adjacent tissues (Fig. 1a). According to circBase (http://www.circbase.org/), we found that circACTN4 was from the ACTN4 gene located on human chromosome (chr) 19, and it was generated from exon 2 to exon 7 of ACTN4 (total 571 bp) by back -splicing, the back splicing junction was verified by Sanger sequencing (Fig. 1b). To verify that circACTN4 is produced by the head to tail splicing rather than trans splicing or genome rearrangement, the convergent and divergent primers were used to amplify circular circACTN4 and linear ACTN4, respectively. RT-PCR revealed that circACTN4 could only be detected in cDNA but not in gDNA, whereas linear ACTN4 was amplified from both cDNA and gDNA by the convergent primers. GAPDH was considered as the reference gene (Fig. 1c). Next, the subcellular localization of circACTN4 was observed with FISH and nuclear/cytoplasmic fractionation assays. The results showed that circACTN4 was predominantly located in the nucleus of BC cells and the expression of circACTN4 in carcinoma tissues was higher than that in adjacent tissues (Fig. 1d and e). Moreover, qRT-PCR and RT-PCR analysis after actinomycin D treatment indicated that circACTN4 was resistant at the indicated time points, whereas linear ACTN4 mRNA transcripts were rapidly degraded (Fig.1f and g). Similarly, We further showed that circACTN4 transcripts was more stable than linear ACTN4 and GAPDH transcripts in response to RNase R digestion with qRT-PCR and RT-PCR (Fig.1h and i). Subsequently, we predicted that upstream transcription factor 2 (USF2) might bind to the promoter of ACTN4 through bioinformatics (http://jaspar.genereg.net). Thus, we inferred that USF2 could regulate the expression of ACTN4 at transcriptional level, and then we constructed luciferase reporter gene vectors carrying the full-length wt (wild type) or mut (mutant) ACTN4 promoter, the results illustrated that USF2 enhanced the luciferase activity of wild type luciferase reporter but not the mutant one (Fig. 1j). In addition, Chip-qPCR assay further proved that USF2 could bind to the ACTN4 gene promoter and enhance its transcriptional activity (Fig. 1k). Hence, the results suggested that USF2 might promote the transcriptional activity of ACTN4. Besides, to further investigate the effects of USF2 on the expression of circACTN4 in BC, we designed and constructed USF2 overexpression and knockdown plasmids, and the transfection efficiency was verified by qRT-PCR (Additional file 2: Fig. S1a). We detected that the expression of ACTN4 in BC cells transfected with the corresponding plasmids by qRT-PCR. The results showed that up-regulated USF2 markedly increased expressions of linear ACTN4 and circACTN4, whereas down-regulated USF2 significantly reduced levels of linear ACTN4 and circACTN4 (Fig. 1l and m). These results suggest that ACTN4 is the target gene of transcription factor USF2 .

### circACTN4 is highly expressed in BC tissues and cells and correlated with clinicopathological features

**Fig. 2:**
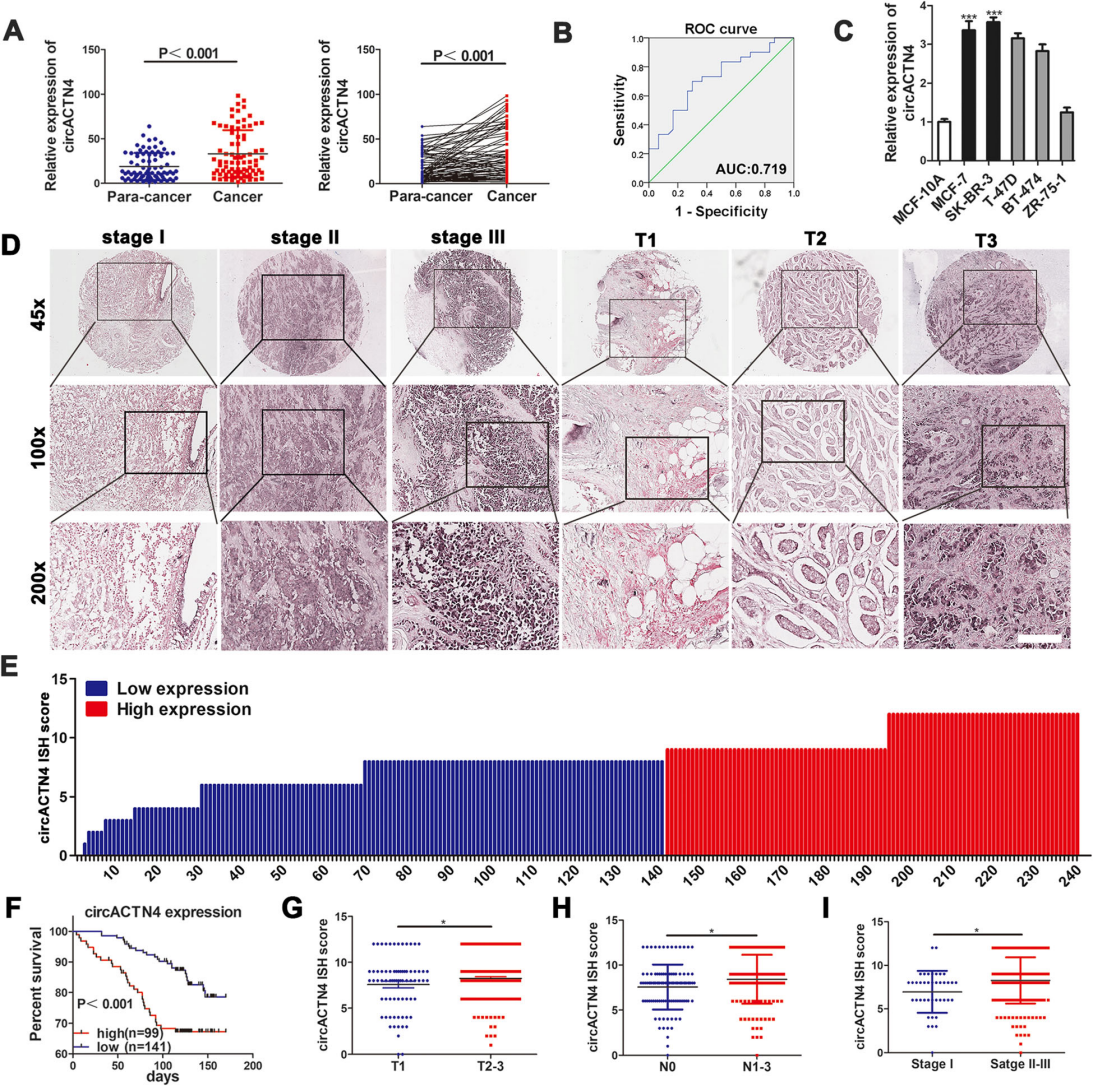
circACTN4 is up-regulated in BC and correlated with progression and poor prognosis of BC patients. **a** The relative expression of circACTN4 was determined in 80 pairs of BC tissues and para-cancer tissues by qRT-PCR. **b** ROC curve was used to assess the diagnostic value of circACN4 for BC. **c** The relative expression of circACTN4 was examined in BC cell lines by qRT-PCR. **d** Representative images indicated the expression of circACTN4 in 240 BC tissues detected with in situ hybridization. Scale bar, 200 μm. **e** The ISH staining scores were calculated in 240 BC tissues. ISH staining score ≤ 8 was regards as low expression, while a score >8 was defined as high expression. **f** Kaplan-Meier survival curve analysis showed that BC patients with high expression of circACTN4 had shorter overall survival time than that of BC patients with low expression of circACTN4. **g-i** Dot distribution graphs of circACTN4 IHC staining scores were shown in 240 BC patients at different clinical stages. GAPDH was used as the normalizing gene in the above experiments. The data are presented as the mean ± SD, *P < 0.05, ***P < 0.001

To probe the expression of circACTN4 and its clinical significance, qRT-PCR was used to detect the expression of circACTN4 in 80 pairs of BC tissues and adjacent non-tumor tissues. The data showed that the expression of circACTN4 in BC tissues was significantly higher than the paired adjacent tissues (Fig. 2a), which was consistent with the results of microarray analysis. And the expression of linear ACTN4 in 20 pairs of BC and para-cancer tissues were detected by qRT-PCR, data showed that linear ACTN4 was highly expressed in BC tissues (Additional file 2: Fig. S1g). Pearson correlation analysis displayed that the level of circACTN4 was positively related with the expression of linear ACTN4 in BC tissues (Additional file 2: Fig. S1h). Results suggest that the co-overexpression of circACTN4 and ACTN4 cooperatively promotes the progression of breast cancer. ROC (Receiver operating characteristic) curve was used to assess the diagnostic efficacy of circACTN4 in BC screening. The AUC (area under the curve) of circACTN4 was 0.719, the diagnostic specificity and sensitivity were 70% and 70 respectively at cut-off value of 12.77 according to the ROC curve analysis (Fig. 2b). Moreover, the expression of circACTN4 was significantly higher in BC cells (MCF-7, SK-BR-3, ZR-75-1, T-47D and BT-474) than normal breast epithelial cell MCF-10A (Fig. 2c). Next, we assessed the association of the expression of circACTN4 with clinicalpathological features. The data revealed that the expression of circACTN4 was positively related with age (*P* = 0.036), T (*P* = 0.001), N (*P* = 0.001) and TNM stages (*P* < 0.001) but not with grade (Table 1). Subsequently, we utilized human tissue microarrays (TMAs) containing 240 BC tissues to determine the expression of circACTN4 by ISH (Fig. 2d and e). The patients with high expression of circACTN4 had a shorter overall survival than that with low expression of circACTN4 by Kaplan-Meier survival analysis (Fig. 2f). The correlation between clinical pathological characteristics and circACTN4 expression of the 240 BC patients was listed in Table 2. Further analysis indicated that circACTN4 expression was positive correlated with T stage, N stage and TNM stage (Fig. 2g-i). The prognostic value of circACTN4 was evaluated by Cox proportional hazard model. The results showed that overexpression of circACTN4 was an independent predictor of poor prognosis in BC patients (HR = 4.566, *P* <0.001) (Table 3). In brief, circACTN4 could play an oncogenic role and high expression of circACTN4 could predict poor prognosis in BC patients.

**Table 1:** Correlation between circACTN4 expression and clinicopathological features in 80 BC patients (cohort 1)

Characteristic		All cases	CircACTN4		Chi-square	*P* value
			low	high		
All cases		80	18	62		
Age	< 53 ≥53	45 35	14 4	31 31	4.374	0.036^*^
Grade	II III	17 63	5 13	12 50	0.591	0.442
T stage	T1 T2–3	35 45	14 4	21 41	10.928	0.001^**^
N stage	N0 N1–3	40 40	15 3	25 37	10.323	0.001^**^
TNM stage	I II/III	23 57	14 4	9 53	27.254	<0.001^***^

^*^P< 0.05,^**^P< 0.01,^***^P< 0.001

**Table 2:** Correlation between circACTN4 expression and clinicopathological features in 240 BC patients (cohort 2)

Characteristic		All cases	CircACTN4		Chi-square	*P* value
			low	high		
All cases		240	141	99		
Age	< 53 ≥53	118 122	68 73	50 49	0.121	0.728
Grade	II III	190 50	114 27	76 23	0.588	0.443
T stage	T1 T2–3	73 167	50 91	23 76	4.109	0.043^*^
N stage	N0 N1–3	108 132	76 65	32 67	10.941	0.001^**^
TNM stage	I II/III	40 200	30 111	10 89	5.230	0.022^*^

^*^P < 0.05, ^**^P < 0.01

**Table 3:** Univariate and multivariate Cox regression analysis of circACTN4 and survival in 240 BC patients

Clinical variables	Univariate analysis		*P*	Multivariate analysis		*P*
	HR	95%CI		HR95%CI		
Age (≥53 vs. < 53)	1.757	1.079–2.860	0.023*	1.894	1.161–3.089	0.011^*^
Grade (II vs. III)	1.982	1.192–3.294	0.008**	1.989	1.194–3.316	0.008^**^
T stage (T1 vs. T2/3)	1.383	0.800–2.392	0.246			
stage (N0–1 vs. N2–3)	2.234	1.327–3.758	0.002**	1.499	0.868–2.591	0.147
TNM stage (I vs. II/III)	2.937	1.182–7.299	0.020*	1.972	0.766–5.077	0.159
CircACTN4 (low vs. high)	5.040	2.913–8.720	<0.001***	4.566	2.612–7.982	<0.001^***^

Abbreviations: *HR* hazard ratio, *CI* confidence interval

^*^P< 0.05, ^**^P< 0.01, ^***^P< 0.001

### circACTN4 enhances proliferation and regulates apoptosis of BC cells

**Fig. 3:**
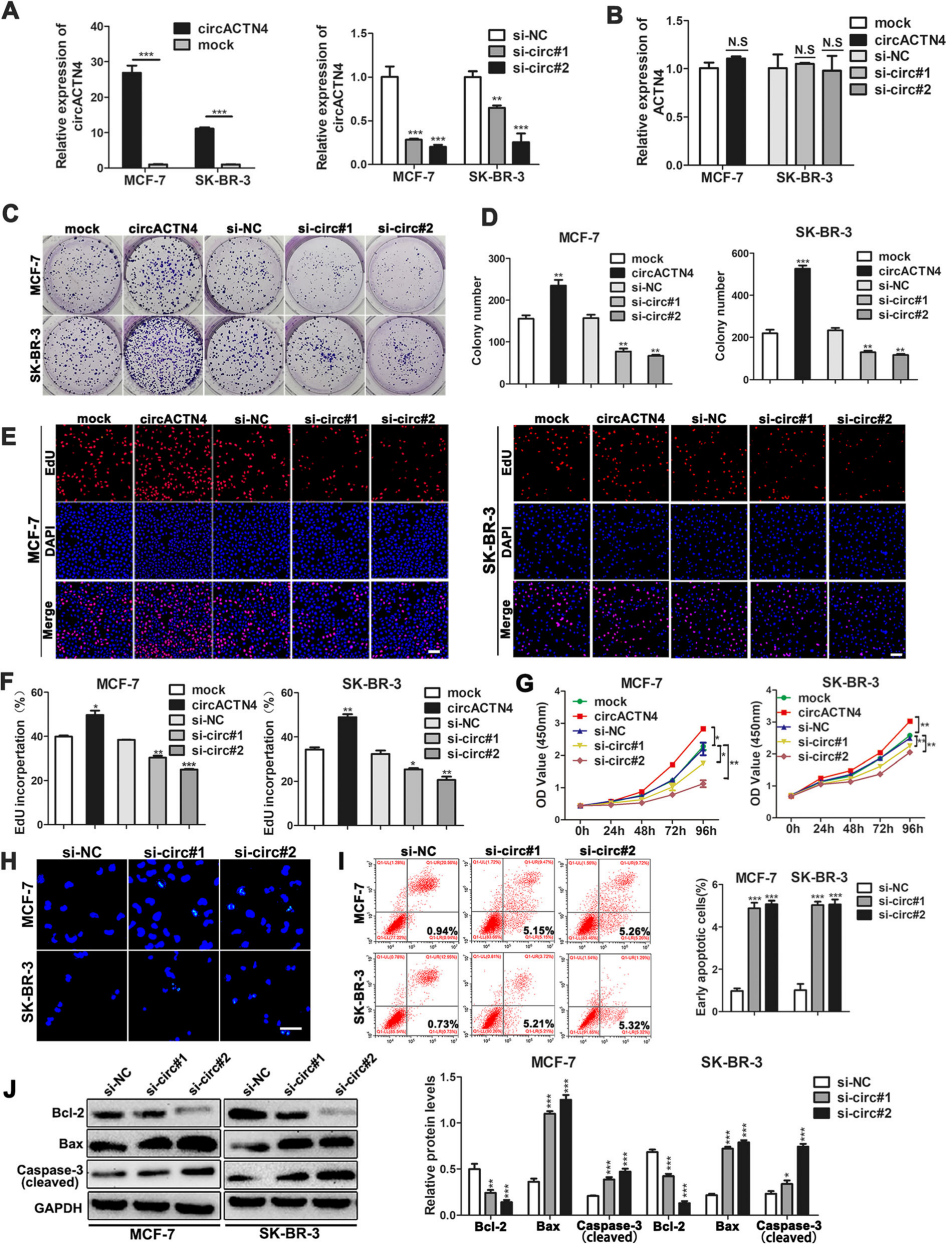
circACTN4 increases proliferation and suppresses apoptosis of BC cells. **a** and **b** The relative expressions of circACTN4 and ACTN4 were detected in BC cells after transfection with overexpressed circACTN4 plasmid or si-circACTN4 by qRT-PCR. **c** and **d** Colony formation assay was used to assess cell survival in BC cells transfected with indicated plasmids and siRNA. **e** and **f** EdU assay was conducted to detect cell proliferation ability of BC cells (magnification, × 100, Scale bar, 100um). **g** CCK-8 assay was performed to evaluate cell viability. **h** Morphological changes of apoptotic cells were observed by Hoechst 33342 staining (magnification,× 400, Scale bar, 50um) after knockdown of circACTN4. **i** The apoptosis rate was analyzed by flow cytometry after downregulation of circACTN4. **j** The expressions of apoptosis-related proteins were determined in BC cells transfected with indicated siRNA by Western blot. GAPDH was used as the normalizing gene in the above experiments. The data are presented as the mean ± SD, *P < 0.05, **P < 0.01, ***P < 0.001

To explore the biological function of circACTN4 in BC cells, circACTN4 overexpression plasmid and small interference RNAs (siRNAs) targeting circACTN4 were transfected into BC cells. The results indicated that the expression of circACTN4 was remarkably up-regulated or down-regulated in BC cells transfected with the corresponding vectors or siRNAs by qRT-PCR (Fig. 3a). However, both overexpression and knockdown of circACTN4 did not impact on the expression level of linear transcript ACTN4 with qRT-PCR (Fig. 3b). The colony formation, EdU and CCK-8 assays showed that overexpression of circACTN4 markedly enhanced the proliferation ability of BC cells, whereas knockdown of circACTN4 significantly inhibited cell viability (Fig. 3c-g). Moreover, hoechst 33342 staining showed that BC cells appeared obvious morphological feature of apoptosis including stronger fluorescenc, enuclear fragment, chromatin aggregation and apoptosis body after transfected with si-circACTN4 (Fig. 3h). In addition, the apoptosis rate was detected by flow cytometry using AnnexinV/PI-staining, and the results indicated that circACTN4 knockdown could induce apoptosis of BC (Fig. 3i). Finally, we detected the apoptosis-related proteins by western blot, results showed that down-regulation of circACTN4 led to higher levels of activated (cleaved) caspase-3 and proapoptotic protein Bax and a lower expression of Bcl-2 compared with control group (Fig. 3j).

### circACTN4 increases migration and invasion and modulates cell cycle progression of BC cells

**Fig. 4:**
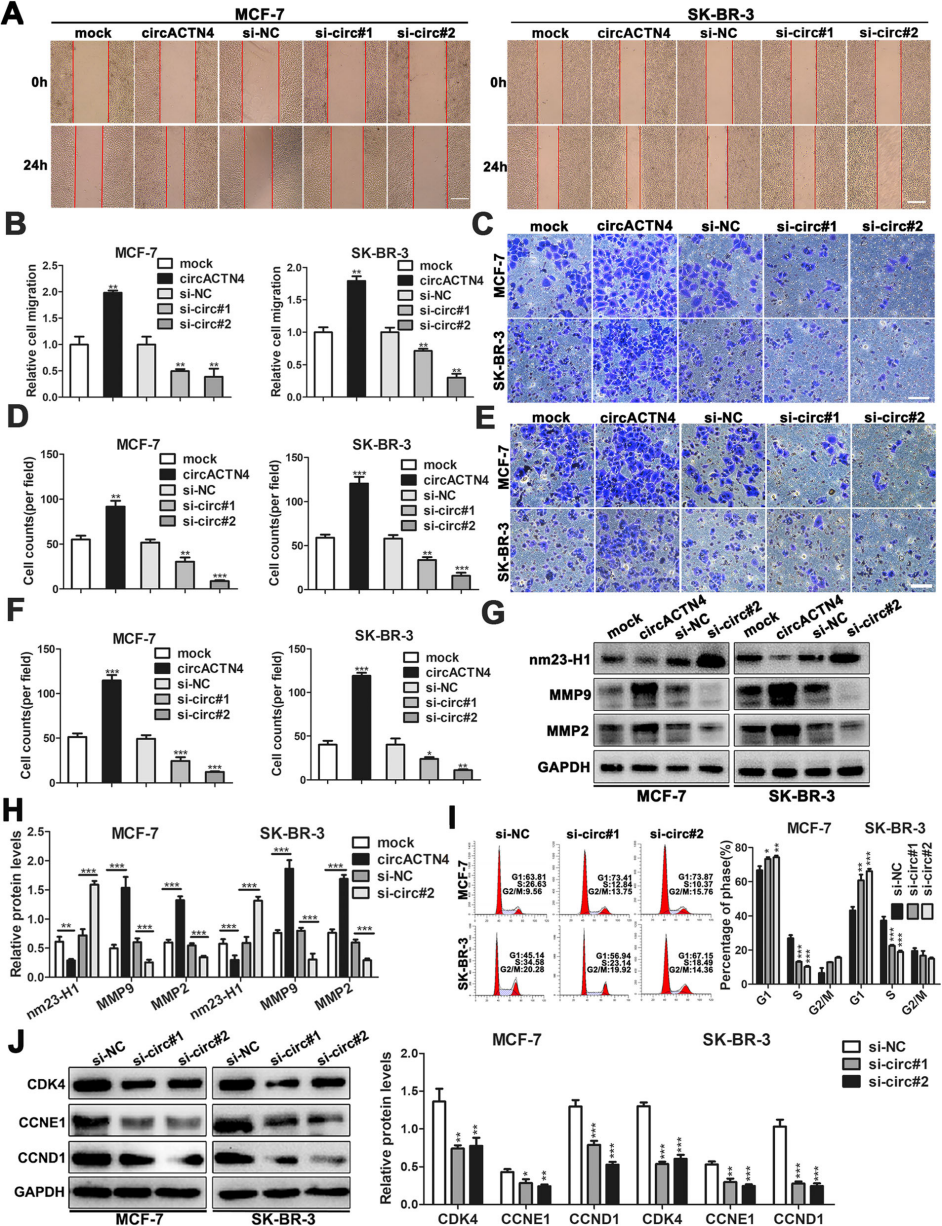
circACTN4 promotes migration, invasion and cell cycle progression of BC cells. **a** and **b** The migration ability of BC cells were detected by wound healing assays (magnification, × 50, Scale bar, 200um) after overexpression or knockdown of circACTN4. **c-f** Transwell assay was used to measure BC cells invasion and migration abilities after upregulation and downregulation of circACTN4, respectively (magnification, × 100, Scale bar, 100 um) . **g and h** The expressions of nm23-H1, MMP9 and MMP2 were detected in BC cells transfected with circACTN4 overexpression plasmids and si-circ#2 by western blot. **i** Cell cycle analysis was executed by flow cytometry after circACTN4 silencing. **j** The expressions of cell cycle-related proteins were detected in BC cells transfected with indicated siRNA by western blot. GAPDH was used as the normalizing gene in the above experiments. The data are presented as the mean ± SD, *P < 0.05,**P < 0.01, ***P < 0.001

To further assess the role of circACTN4 in BC progression, migration, invasion and cell cycle of BC cells were investigate after overexpression or knockdown of circACTN4. The wound healing and transwell assays indicated that the invasive and migratory abilities of BC cells were remarkably increased by up-regulation of circACTN4 but significantly suppressed by down-regulation of circACTN4 (Fig. 4a-f). Furthermore, the expressions of nm23-H1, MMP9 and MMP2 were detected by western blot, the results showed that MMP9 and MMP2 protein levels were obviously enhanced or reduced and nm23-H1 expression was markedly decreased or increased in BC cells after overexpression or knockdown of circACTN4 (Fig. 4g and h). Moreover, cell cycle analysis was determined by flow cytometry, the results revealed that knockdown of circACTN4 led to lower percentage of BC cells in S phase and higher proportion of BC cells in G1 phase compared with control group, which suggest that circACTN4 silencing caused G1 arrest of BC cells (Fig. 4i). Additionally, the expression of cell cycle- related proteins was measured with western blot, we found that CDK4, CCNE1 and CCND1 protein levels were significantly downregulated after knockdown of circACTN4 in BC cells, which might prevent cell cycle progression of BC cells (Fig. 4j).

### CircACTN4 directly interacts with FUBP1 and activates transcription of MYC

**Fig. 5:**
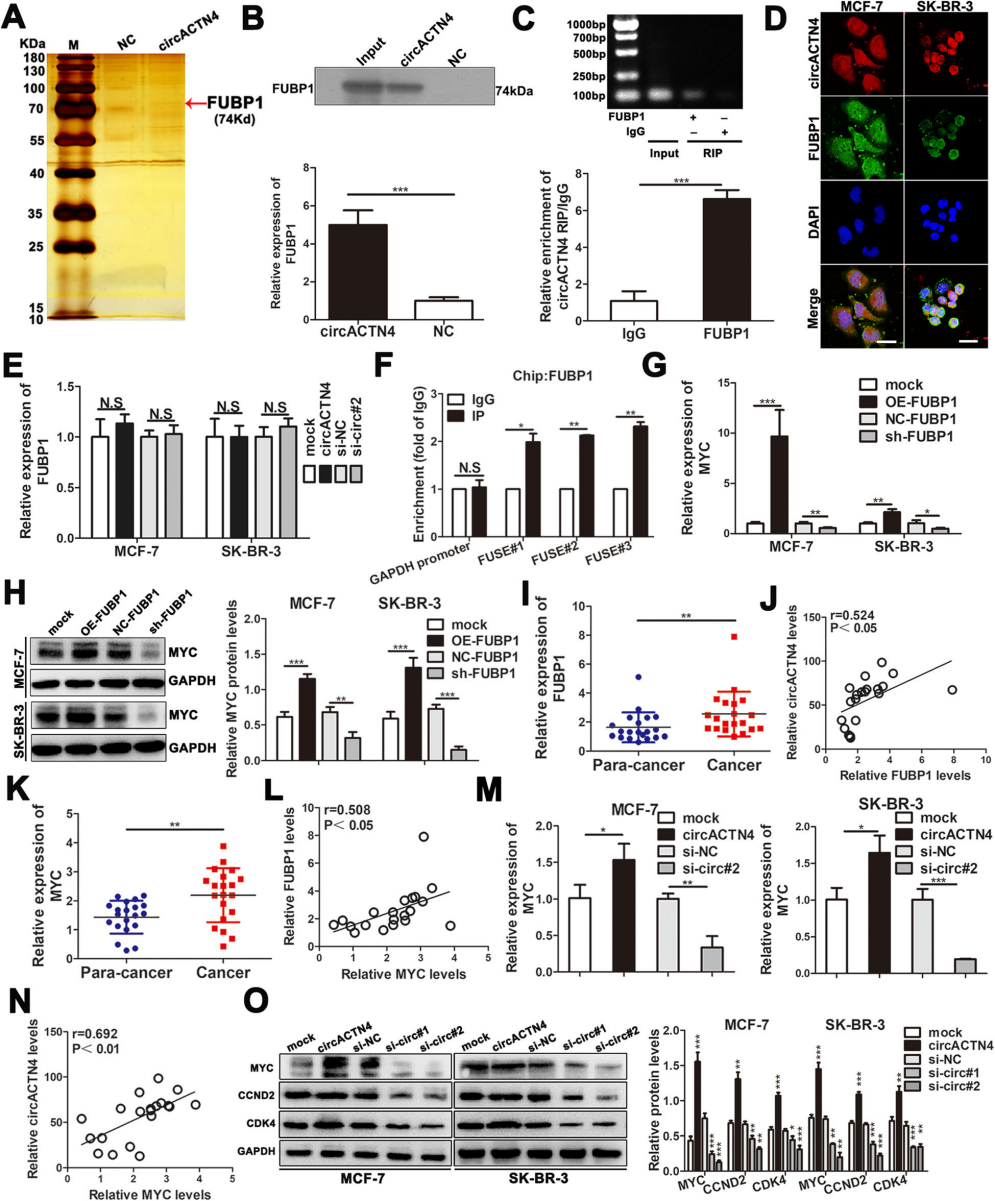
circACTN4 can bind to FUBP1 to upregulate MYC expression. **a** and **b** RNA pull-down experiment was performed using the specific biotin-labeled circACTN4 probe in MCF-7 cell lysates, followed by silver staining, and the protein bands were analyzed by mass spectrometry and western blot. FUBP1 was identified as a candidate protein interacting with circACTN4. **c** RIP assay was executed in MCF-7 cell lysates using anti-FUBP1or anti-IgG, then the enrichment of circACTN4 was detected by RT-PCR and qRT-PCR. **d** FISH-IF assay displayed that circACTN4 was co-localized with FUBP1 in the nucleus of BC cells (magnification, × 1000, Scale bar, 25um). **e** The relative expression of FUBP1 was determined in the BC cells transfected with overexpressed circACTN4 plasmid or si-circACTN4 by qRT-PCR. **f** Chip assay showed that FUBP1 was enriched in the FUSE upstream of MYC promoter and was not enriched in the GAPDH promoter. **g** and **h** The effects of overexpression and knockdown of FUBP1 in BC cells on the expression of MYC were detected by qRT-PCR and western blot. **i** The relative expression of FUBP1 in 20 pairs of BC tissues and para-cancer tissues was determined by qRT-PCR. **j** Pearson correlation analysis showed that the expression of FUBP1 was positively correlated with the level of circACTN4. **k** The relative expression of MYC in 20 pairs of BC tissues and para-cancer tissues was detected by qRT-PCR. **l** Pearson correlation analysis showed that the expression of FUBP1 was positively correlated with that of MYC. **m** The relative expression of MYC was evaluated after transfection with overexpressed circACTN4 plasmid and si-circACTN4 by qRT-PCR. **n** The expression of circACTN4 was positively associated with that of MYC by pearson correlation analysis. **o** The expressions of MYC and its downstream proteins CDK4 and CCND2 were assessed in the BC cells transfected with overexpressed circACTN4 plasmid and si-circACTN4 by western blot. GAPDH was used as the normalizing gene in the above experiments. The data are presented as the mean ± SD, *P < 0.05, **P < 0.01, ***P < 0.001

To explore the molecular mechanism underlying circACTN4, we first performed pull down, followed by silver staining and mass spectrometry analysis to detect the circACTN4-binding proteins, we found that FUBP1 was significantly enriched on circACTN4 (Fig. 5a and b). Subsequently, RIP assay further confirmed that FUBP1 could specifically combine with endogenous circACTN4 in BC cells by PCR and qRT-PCR (Fig. 5c). FISH-IF assay displayed that circACTN4 and FUBP1 were co-localized in the nucleus (Fig. 5d). But upregulation and knockdown of circACTN4 did not alter FUBP1 expression levels (Fig. 5e), suggesting that circACTN4 was not involved in the post-translational regulation of FUBP1. Previous studies have shown that FUBP1 could bind to the FUSE region of the MYC promoter to promote the expression of MYC, while FUBP1-FUSE complex might recruit FIR to inhibit MYC transcription. Next, ChIP-PCR assay confirmed that FUBP1 binds to FUSE of the MYC promoter in BC cells (Fig. 5f). To explore the effects of FUBP1 and FIR in BC, we constructed FUBP1 and FIR overexpression and knockdown plasmids. The transfection efficiencies were determined by qRT-PCR and western blot. (Additional file 2: Fig. S1b-e). The results of qRT-PCR and western blot demonstrated that upregulation or downregulation of FUBP1 evidently increased or decreased expression levels of MYC in BC cells, respectively (Fig. 5g and h). Moreover, we found that the expression of FUBP1 in BC tissues was noticeably higher than that in adjacent normal tissues by qRT-PCR (Fig. 5i). Pearson correlation analysis showed that the level of circACTN4 was positively related with the expression of FUBP1 in BC tissues (Fig. 5j). Furthermore, MYC was highly expressed in BC tissues and positively correlated with the expression of FUBP1 (Fig. 5k and l). We also demonstrated that there was a positive correlation between the expression of circACTN4 and MYC by qRT-PCR and Pearson correlation analysis (Fig. 5m and n). In addition, some studies have shown that ACTN4 can also promote the progression of breast cancer. To confirm the biological function of ACTN4 in BC, ACTN4 overexpression and interference plasmids were constructed, the transfection efficiencies were determined by qRT-PCR (Additional file 2: Fig. S1i). And then, to probe that the effect of circACTN4 is independent of ACTN4, co-transfection experiments of circACTN4 or si-circ#2 and sh-ACTN4 or OE-ACNT4 were conducted, the results showed that ACTN4 knockdown did not affect the promoting role of overexpressed circACTN4 on MYC transcription, and upregulation of ACTN4 did not reverse the inhibitory effect of circACTN4 knockdown on MYC expression by qRT-PCR and western blot (Additional file 2: Fig. S1j and k). Additionally, the results of western blot showed that circACTN4 could promote the expressions of MYC and its downstream proteins CDK4 and CCND2 (Fig. 5o). However, whether circACTN4 was up-regulated or down regulated in BC cells, there was no effect on the protein levels of FUBP1 and FIR (Additional file 2: Fig. S1f). Together, these data suggest that circACTN4 could bind to FUBP1 and promote the expression of MYC.

### circACTN4 and FIR competitively combine with FUBP1

**Fig. 6:**
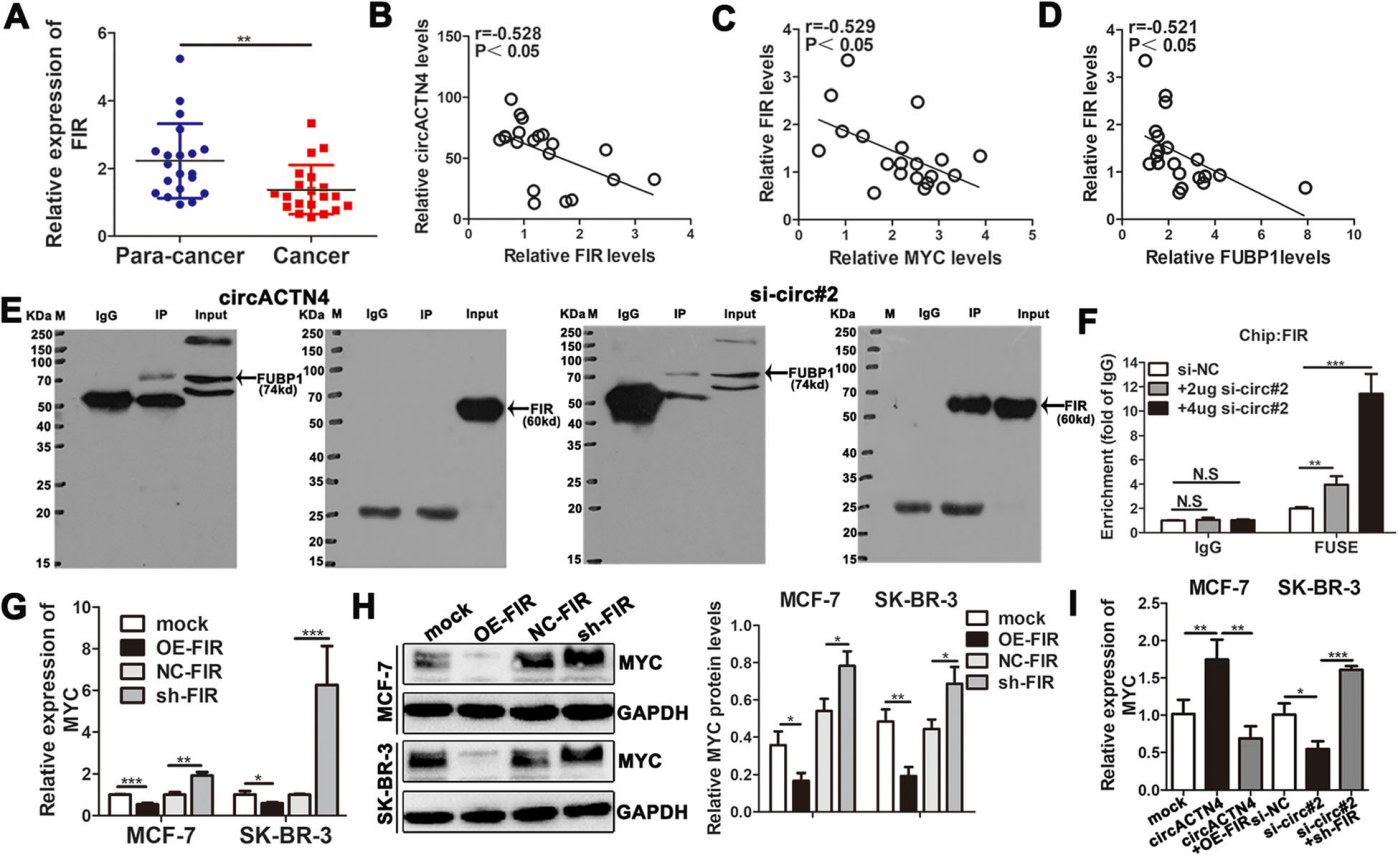
circACTN4 blocks interaction FIR with FUBP1. **a** The relative expression of FIR in 20 pairs of BC tissues and para-cancer tissues was determined by qRT-PCR. **b -d** Pearson correlation analysis showed that the expression of FIR was negatively correlated with those of circACTN4, MYC and FUBP1. **e** Co-IP assay showed that FIR could not be pulled down by anti-FUBP1 in MCF-7 cells with transfected with overexpressed circACTN4 plasmid but FIR could be detected in MCF-7 cells with transfected with si-circACTN4. **f** Chip assay showed that the transfection of BC cells with different concentrations of si-circ#2 affected the binding of FIR and FUSE. **g** and **h** The effects of knockdown and overexpression of FIR in BC cells on the expression of MYC were determined by qRT-PCR and western blot. **i** The expression of MYC was detected after transfection or co-transfection with the indicated vectors and siRNA by qRT-PCR. GAPDH was used as the normalizing gene in the above experiments. The data are presented as the mean ± SD, *P < 0.05,**P < 0.01, ***P < 0.001

Therefore, we presume that circACTN4 could competitively combine with FUBP1 and prevent the binding of FIR to FUBP1 to promote the transcription and expression of MYC. In order to further verify proposed hypothesis, we first detected the expression of FIR in BC issues and matched normal tissues by qRT-PCR, and our data revealed that the expression of FIR was significantly downregulated in BC tissues compared with normal adjacent tissues (Fig. 6a). Pearson correlation analysis indicated that the expression of FIR was negatively associated with the levels of circACTN4, FUBP1 and MYC in BC tissues (Fig. 6b-d). Subseqently, we explored whether circACTN4 could influence the binding between FIR and FUBP1. Co-immunoprecipitation assays were performed in MCF-7 cells with anti-FUBP1 antibody. The results showed that no obvious FIR protein band was found in the FUBP1-co-immunocoprecipitates of BC cells overexpressing circACTN4, whereas FIR was clearly detected in the co-immunocoprecipitates by western blot after circACTN4 was knocked down, which suggest upregulated circACTN4 remarkably weakened the binding of FIR to FUBP1 while downregulated circACTN4 significantly strengthened the interaction between FUBP1 and FIR (Fig. 6e). A series of ChIP assay results showed that the binding level of FIR and FUSE after transfection with high concentration of si-circ#2 was higher than that of FIR and FUSE after transfection with low concentration of si-circ#2 (Fig. 6f), but had no effect on the binding level of FUBP1 and FUSE after transfection with high or low concentration of si-circ#2 (Additional file 2: Fig. S1l). Furthermore, we found that silencing FIR markedly enhanced the expression of MYC, whereas overexpressing FIR decreased the level of MYC by qRT-PCR and western blot in BC cells (Fig. 6g and h). Finally, after co-transfection of OE-FIR or sh-FIR with circACTN4 or si-circ#2, we found that overexpression FIR could reverse the enhancing effect of circACTN4 on MYC expression, while FIR knockdown could counteract the inhibitory impact of downregulation of circACTN4 on MYC level by qRT-PCR (Fig. 6i). In summary, these results demonstrated that circACTN4 could inhibit the binding FIR and FUBP1 to relieve the repressive effect of FIR, thereby promoting MYC transcription.

### FIR reverses the tumor promoting-effects of circACTN4 in BC cells

**Fig. 7:**
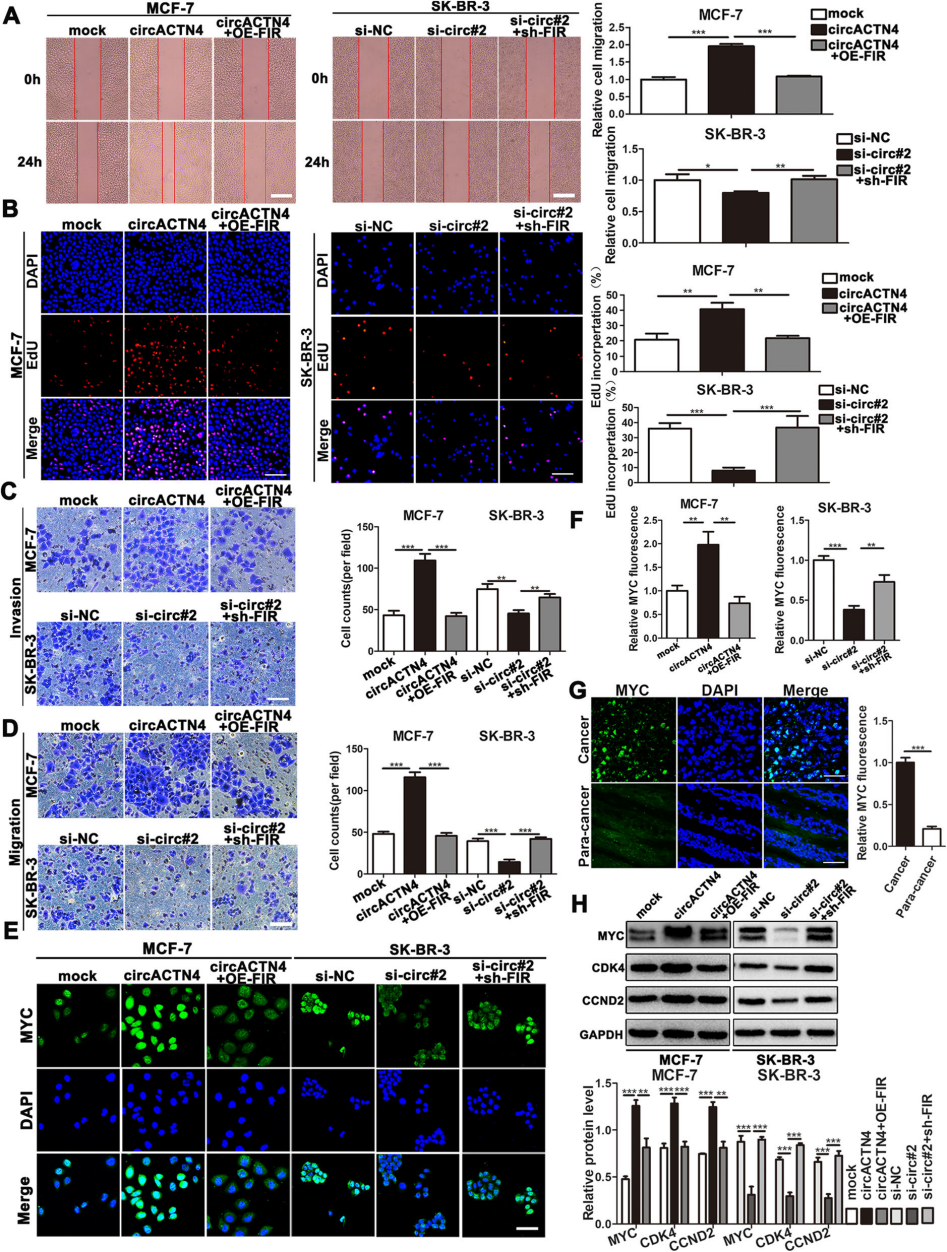
FIR reverses the oncogenic effects of circACTN4 in BC cells. **a** The ability of cell migration was detected by wound healing assay after transfection or co-transfection with indicated vectors or si-circ#2 (magnification, × 50, Scale bar, 200 um) . **b** The ability of cell proliferation was evaluated after transfection and co-transfection with circACTN4 plasmid, si-circACTN4, OE-FIR or sh-FIR vectors by EdU (magnification, × 100, Scale bar, 100 um) assay. **c** and **d** The invasion and migration abilities of BC cells transfected or co-transfected with circACTN4 plasmid, si-circACTN4, OE-FIR or sh-FIR vectors were measured by transwell invasion and transwell migration (magnification, × 100, Scale bar, 100 um), respectively. **e and f** The expression of MYC in BC cells transfected or co-transfected with indicated vectors and siRNA was detected by IF (magnification, × 200, Scale bar, 200 um). **g** The expression of MYC in breast cancer and paracancer tissues was displayed by IF (magnification, × 400, Scale bar, 50 um). **h** The expressions of MYC and its downstream proteins CDK4 and CCND2 were detected after transfection and co-transfection with indicated vectors and siRNA by western blot. GAPDH was used as the normalizing gene in the above experiments. The data are presented as the mean ± SD, *P < 0.05,**P < 0.01, ***P < 0.001

To further investigate whether circACTN4 plays its biological role via circACTN4/FUBP1/MYC axis, a series of rescue experiments were executed in BC cells after co-transfection of OE-FIR or sh-FIR with circACTN4 or si-circ#2. The results show that FIR overexpression could markedly reverse the promoting roles of circACTN4 upregulation in proliferation, migration and invasion of BC cells, while FIR knockdown attenuated the inhibitory effects mediated by circACTN4 downregulation in BC cells by wound healing, EdU and transwell assays (Fig. 7a-d). Moreover, IF assay also showed that FIR overexpression and knockdown could evidently reverse the effects of upregulation and downregulation of circACTN4 on MYC expression (Fig. 7e and f). Additionally, compared with para-cancer tissues, we found that MYC was highly expressed in BC tissues by IF (Fig. 7g). Furthermore, western blot analysis showed that upregulation and downregulation of FIR could counteract the enhancing and suppressing effects on the expression of MYC and its downstream protein CDK4 and CCND2 by overexpressing and silencing circACTN4 (Fig. 7h). Collectively, the above results further proved that circACTN4 might competitively bind with FUBP1 to block the transcriptional inhibitory effect of FIR on MYC, which could promote to tumorigenesis and progression of BC.

### CircACTN4 facilitates tumorigenesis and metastasis of xenograft tumors in vivo

**Fig. 8:**
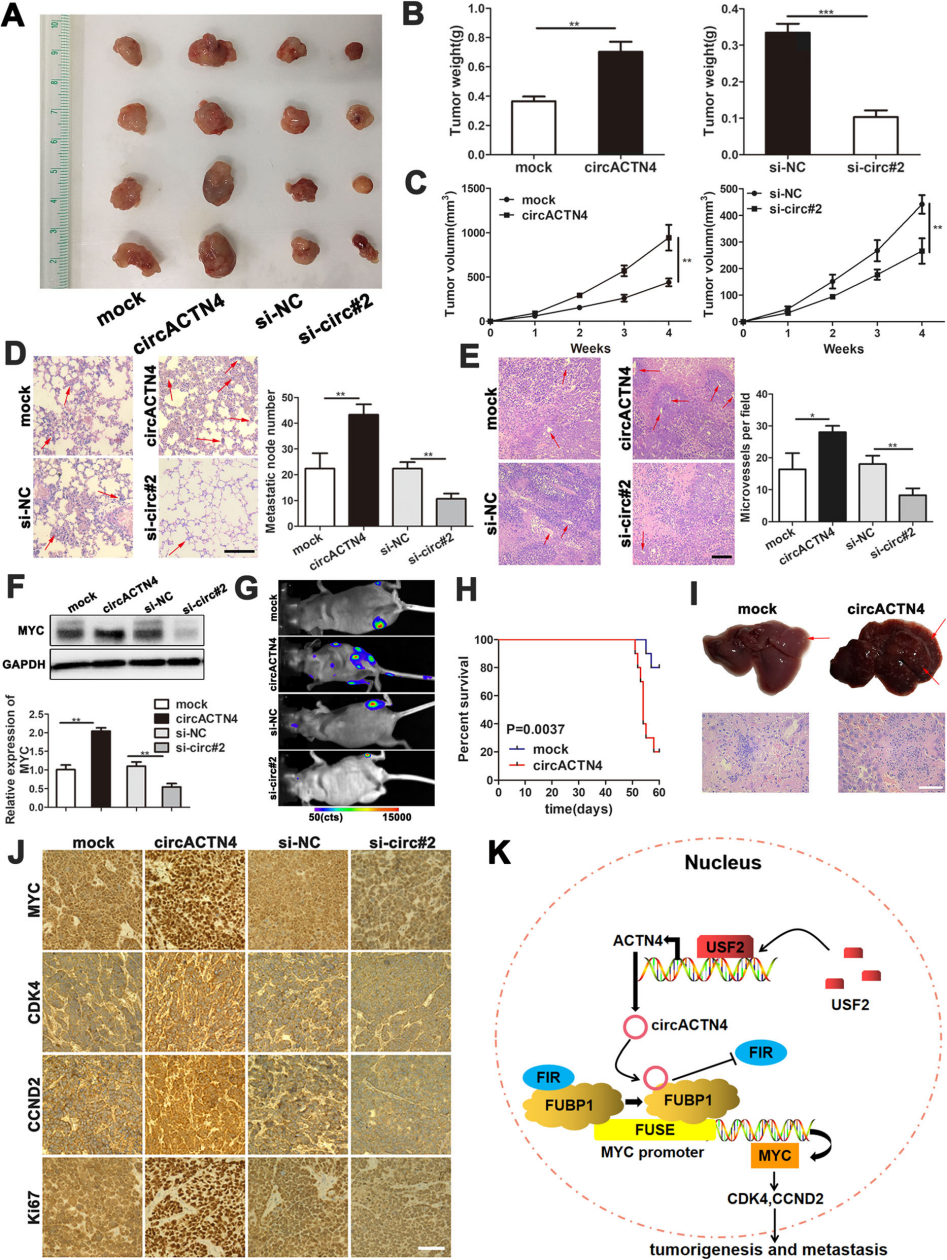
circACTN4 promotes tumorigenesis and metastasis of BC cells. **a** and **b** Representative images of xenograft tumors of each group and tumor weight analysis were shown. **c** The tumor volumes were measured once a week and the growth curves were drawn. **d** and **e** HE staining of lung and tumor sections were showed. Metastatic nodules of the lungs and microvessels of the tumors were analyzed and indicated with arrows (magnification, × 100, Scale bar, 100 μm). **f** Western blot analysis was used to detect the protein level of MYC in xenograft tumor tissues. **g** Representative bioluminescent imaging (BLI) of the different groups was shown. circACTN4 knockdown and overexpression MCF-7 cells were injected into tail vein of mice for 4 weeks to detect metastasis (*n* = 5 for each group). **h** The survival curve was generated for the nude mice injected with MCF-7 cells infected with overexpressed circACTN4 or mock vector using Kaplan–Meier survival analysis (*n* = 10 for each group). **i** The representative images of liver metastasis were taken from nude mice injected with circACTN4 overexpressing and mock control MCF-7 cells for 60 days (magnification, × 400, Scale bar, 100 μm). **j** The expressions of MYC, CDK4, CCND2 and Ki67 in tumors of nude mice were detected by immunohistochemistry (magnification, × 200, Scale bar, 200 μm). **k** The schematic diagram shows how circACTN4 could promote tumorigenesis and metastasis of BC through circACTN4/FUBP1/MYC axis. GAPDH was used as the normalizing gene in the above experiments. The data are presented as the mean ± SD, *P < 0.05,**P < 0.01, ***P < 0.001

To evaluate the oncogenic effect of circACTN4 in vivo, the tumor models of human BC cells were established in nude mice by subcutaneous injection and caudal vein injection. Female nude mice were inoculated with the MCF-7 cells infected with circACTN4 overexpressing lentiviruses or circACTN4 shRNA lentiviruses and their controls. The results indicated that the volume and weight of xenograft tumor in overexpression circACTN4 group were markedly larger than those in the control group, whereas circACTN4 knockdown significantly suppressed the tumor growth (Fig. 8a-c). Furthermore, compared with control group, the upregulation of circACTN4 obviously increased the number of metastatic pulmonary nodules, while circACTN4 silencing significantly repressed spontaneous pulmonary metastasis with less invasive tumor cells (Fig. 8d). Moreover, the overexpression of circACTN4 could remarkably facilitate tumor angiogenesis, whereas circACTN4 knockdown markedly reduced tumor microvessel density (Fig. 8e). Next, the results of western blotting revealed that the protein level of MYC in the xenograft tumor tissue from overexpressing or silencing circACTN4 group was significantly increased or decreased, respectively compared with the control group (Fig. 8f). Subsequently, to further observe the impact of circACTN4 on tumor metastasis in vivo, stable circACTN4-overexpressing and circACTN4-silencing MCF-7 cells were intravenously injected into the tail vein of BALB/c nude mice. The micrometastases were monitored using bioluminescent imaging (BLI). The results displayed that stronger and more bioluminescent signals were detected in the circACTN4 overexpressing group, while mice in circACTN4 silencing group displayed smaller the number of bioluminescent metastases compared with the control group (Fig. 8g). In addition, the mice in the circACTN4 overexpressing group had lower overall survival rate and more extensive liver metastasis compared with the control group (Fig. 8h and i). Finally, we determined the effects of circACTN4 on its target protein MYC, downstream cell cycle related proteins CDK4 and CCND2 as well as cell proliferation related protein Ki67 by IHC. The results showed that upregulation of circACTN4 could increase the expressions of MYC, CDK4, CCND2 and Ki67 in tumor tissues, whereas circACTN4 silencing reduced the expression levels of these proteins (Fig. 8j). Together, these above results further demonstrated the oncogenic role of circACTN4 in BC, suggesting that circACTN4 could promote development and metastasis of breast cancer through activating proto-oncogene MYC.

## Discussion

Breast cancer is a heterogeneous and complex disease with the high incidence and the mortality in female worldwide. Though some breast cancer patients reveal good prognosis, there are still some patients with tumor progression, which has become a threat to public health [22]. Emerging evidence suggests that circRNAs play pivotal roles in the tumorigenesis and development of diverse human cancers [2]. However, the molecular mechanism of circRNAs in the progression of breast cancer is largely unknown. In this study, we found that circACTN4 (hsa_circ_0050900), a novel nuclear circRNA, was significantly overexpressed in breast cancer and was closely related to advanced clinical stage, metastasis and poor prognosis of BC patients. Functionally, we showed that up-regulation of circACTN4 promoted the growth and aggressiveness of breast cancer cells in vitro and in vivo, while down-regulation of circACTN4 had the contrary effects. Mechanistically, we found that USF2 could accelerate the biogenesis of circACTN4. More importantly, we discovered that circACTN4 could competitively bind to FUBP1 and block the connection between FUBP1 and FIR, thus stimulating MYC transcription. Our study demonstrate that circACTN4 is a novel oncogenic circRNA in breast cancer through activation of MYC and plays an important role in the progression of breast cancer.

Most circRNAs were reported to locate in the cytoplasm and work as miRNA sponge, which regulate tumor progression via competing endogenous RNA (ceRNA) mechanism [23]. However, some circRNAs are predominantly expressed in the nucleus to combine with RNA-binding proteins and regulate their activity. For example, circCUX1 binds with EWS RNA-binding protein 1 (EWSR1) to promote its interplay with MAZ (MYC-associated zinc finger protein), glycolysis and neuroblastoma progression [24]. Furthermore, circ-CTNNB1 binding DEAD-box polypeptide 3 (DDX3) promotes its physical interaction with transcription factor Yin Yang 1 (YY1) [25]. Additionally, circ-DONSON might recruit the NURF complex to transcription factor SOX4 promoter and initiate its transcription, thus facilitating gastric cancer growth and invasion [26]. In our study, we revealed that circACTN4 was primarily located in the nucleus of BC cells, which suggested that circACTN4 might be not a miRNA sponge. The function and mechanism of circRNAs are related to their subcellular localization pattern [27]. We demonstrated that circACTN4 was involved in transcription of MYC, which occurs in nucleus and control cell proliferation [28]. Up to now, how circRNAs exert functions in the nucleus remains elusive.

FUBP1, an ATP dependent DNA helicase V, is a significant regulator of transcription, translation and RNA splicing by binding with single stranded DNA (ssDNA) and RNA. FUBP1 binds to the FUSE (far upstream element) of c-myc promoter and activates transcription of c-myc by TFIIH helicase activity. FIR could inhibit the activation effect of FUBP1. The interaction of FUBP1 with FIR and TFIIH affected the time and the level of MYC gene expression. Therefore, FUSE/FUBP1/FIR complex provides a fine-tuned regulation of MYC [29, 30]. Emerging evidence has showed both the oncogenic and suppressive roles of FUBP1. Mutations in the FUBP1 gene and loss of function are frequently found in patients with central nervous system diseases and intestinal cancer [17, 31]. Whereas, overexpression of FUBP1 is also often appears in various cancers including breast cancer, gastric cancer, hepatocellular carcinoma, nasopharyngeal carcinoma and leukemia [19, 32–35]. High expression of FUBP1 usually leads to upregulation of c-MYC oncogene and disorder of the fine regulation of target proteins, which is one of main molecular mechanisms of tumor pathogenesis. For example, there is a positive correlation between the expression of FUBP1 and c-MYC in glioma and high expressions of FBP1 and c-MYC are associated with poor prognosis [36]. FUBP1 stimulates c-MYC expression in esophageal squamous cell carcinoma (ESCC) and facilitates ESCC development [37]. Sun. et al. demonstrated that lncRNA SNHG1 regulated the expression of MYC by interacting with FUBP1 and preventing the binding of FUBP1 and FIR, thus blocking FIR mediated MYC transcription inhibition [20]. Consistent with the previous reports, we found that FUBP1 was highly expressed in BC tissues, and further demonstrated circACTN4 could directly bind to FUBP1, we also found a positive correlation between the expression of circACTN4 and MYC in BC. Besides, we found that the expression of FIR was negatively correlated with expression of MYC. Moreover, upregulated circACTN4 inhibited the combination of FUBP1/FUSE complex and FIR, while downregulated circACTN4 exerted the opposite effect. These results confirmed that circACTN4 might directly interacted with FUBP1 to prevent the binding of FUBP1 to FIR, thus promoting the expression of the oncogene MYC and progression of BC.

MYC play an important oncogenic role in nearly every aspect of biological processes including proliferation, differentiation, apoptosis and metabolism. MYC is frequently deregulated in human cancers and associated with poor prognosis. It is estimated that its expression is up-regulated or deregulated in up to 70% of human cancers [38]. For example, it was reported that the level of plasma c-MYC in breast cancer patients was markedly higher than that of normal control and was related to clinical stage and lymph node status [39]. Moreover, overexpression of MYC protein is a characteristic of progression and poor prognosis in multiple myeloma [40]. Overexpressing c-MYC promotes melanoma metastasis and is obviously related with distant metastasis and poor prognosis [41]. Overexpression of lncRNA EPIC1 is associated with poor prognosis in patients with luminal type B breast cancer, and promotes tumor growth and cell cycle progression by interacting with MYC [42]. In present study, we found that circACTN4 could promote the expression of MYC by the binding of FUBP1 to MYC promoter and MYC was highly expressed in BC tissues. MYC as a transcription factor, its main downstream effectors participate ribosome biogenesis, protein translation, metabolism and cell cycle progression [43]. MYC promotes cell-cycle progression through the regulating various genes related to cell cycle. Among the MYC targets, CCND2 forms a complex with CDK4 or CDK6 and functions as a regulatory subunit of the complex, whose activity is crucial for cell cycle G1/S transition [44, 45]. We showed that circACTN4 could promote the expressions of its downstream targets CDK4 and CCND2 by activating the expression of MYC. Our experimental results support previous findings.

Transcription factor upstream stimulatory factor 2 (USF2) plays an important role in tumorigenesis and development. Interestingly, USF2 has been proven to have a dual function as either tumor-suppressor or tumor-promoter [46]. However, the underlying mechanism of tumor inhibition or promotion remains largely unclear. It was reported that USF2 was abnormally high expressed in human breast cancers and correlated with cancer progression [47]. Consistent with these studies, we found that USF2 might bind to ACTN4 gene promoter by bioinformatics analysis, ChIP and luciferase reporter assays. Furthermore, we revealed that upregulation of USF2 could increase the level of circACTN4, whereas, knockdown of USF2 suppressed the expression of circACTN4, which suggest that USF2 could stimulate ACTN4 gene transcription. Currently, little is known about the regulation mechanisms of upstream transcription factors on circRNA. Our previous research demonstrated that transcription factors E2F1 might promote circSEPT9 gene transcription and enhance circSEPT9 expression [21]. Further research is needed to clarify the exact molecular mechanism for the regulatory process.

## Conclusion

Taken together, we identified a novel circular RNA circACTN4 that was upregulated in human breast cancer tissues and cells, and we found that increased circACTN4 expression was correlated with poor prognosis of breast cancer patients. Furthermore, we demonstrated that overexpression of circACTN4 could effectively increase BC cell proliferation, invasion and metastasis. Mechanistically, we first discovered that circACTN4 mediated by USF2 might directly bind to FUBP1 and their interaction could impede of the binding of FUBP1 with FIR, thereby activating transcription of MYC and promoting BC development. Our findings suggest that circACTN4 could be a novel prognostic biomarker and promising therapeutical target for breast cancer.

## Supplementary Information

**Additional file 1: Table S1.** Primer sequences of qRT-PCR and PCR used in this study. **Table S2.** Sequences of siRNAs and shRNAs used in this study. **Table S3.** Sequences of probes used in this study.

**Additional file 2: Figure S1.** The expression levels of USF2, FUBP1, FIR, ACTN4 and MYC were determined by qRT-PCR or western blot and the binding of FUBP1 and FUSE was detected by CHIP-qPCR. a Relative expression of USF2 in BC cells was evaluated by qRT-PCR after overexpression and knockdown of USF2. b and c The expression of FUBP1 in BC cells was detected by qRT-PCR or western blot after overexpression and knockdown of FUBP1. d and e The expression level of FIR was determined in BC cells after overexpression and knockdown of FIR by qRT-PCR or western blot. f The expression levels of FUBP1 and FIR were detected in BC cells transfected with circACTN4 overexpression plasmids and si-circ#2 by western blot. g Relative expression of ACTN4 was determined in 20 pairs BC tissues and para-cancer tissues by qRT-PCR. h Pearson correlation analysis showed that the expression of ACTN4 was positively correlated with the level of circACTN4 in breast cancer tissues. i Relative expression of ACTN4 was evaluated in BC cells after transfection with ACTN4 overexpression or knockdown plasmids by qRT-PCR. j and k The expression level of MYC was detected after transfection or co-transfection with the indicated vectors and siRNA by qRT-PCR and western blot. l Chip assay showed that the transfection of BC cells with different concentrations of si-circ#2 had no effect on the binding of FUBP1 and FUSE. GAPDH was used as the normalizing gene in the above experiments. The data are presented as the mean ± SD, *P < 0.05, **P < 0.01, ***P < 0.001.

## Data Availability

The datasets used and analyzed during the current study are available from the corresponding author on reasonable request.
