# Supplementary material for: The circACTN4 interacts with FUBP1 to promote tumorigenesis and progression of breast cancer by regulating the expression of proto-oncogene MYC
Source: Mol Cancer. 2021 Jun 11;20:91. doi: 10.1186/s12943-021-01383-x (PMC8194204; doi:10.1186/s12943-021-01383-x)
Supplement: Supplementary file 1 — Additional file 1: Table S1. Primer sequences of qRT-PCR and PCR used in this study. Table S2. Sequences of siRNAs and shRNAs used in this study. Table S3. Sequences of probes used in this study. [file 12943_2021_1383_MOESM1_ESM.docx]

**Table S1. Sequences of primers used in this study.**

| **Gene** | **Primer sequences** |
| --- | --- |
| CircACTN4 | F:5’-AAGGACGACCCTGTCACCAA-3’ |
|  | R:5’-CCGTGAAGGTCCTCTGCAT-3’ |
| GAPDH | F: 5’-GAAGGTGAAGGTCGGAGTC-3’ |
|  | R: 5’-GAAGATGGTGATGGGATTTC-3’ |
| U6 | F: 5’-CTCGCTTCGGCAGCACA-3’ |
|  | R: 5’-AACGCTTCACGAATTTGCGT-3’ |
|  | RT: 5’-CTCGCTTCGGCAGCACAPCR-3’ |
| USF2F**2** | F: 5’-CTGTGATCCAAAATCCCTTCAGC-3’ |
|  | R: 5’-GGTCTGTGGTCTGTACGGAC-3’ |
| FUBP1 | F: 5’-CAACCAGATGCTAAGAAAGTTGC-3’ |
|  | R: 5’-CCTCCTCTGCCAATTATGAATCC-3’ |
| FIR | F: 5’-GCTTCATTGAGTACGAGAAGGC-3’ |
|  | R: 5’-GCGTGGCTGGTGTGAGTAG-3’ |
| MYC | F: 5’-GGCTCCTGGCAAAAGGTCA-3’ |
|  | R: 5’-CTGCGTAGTTGTGCTGATGT-3’ |
| ACTN4 | F: 5’-ACCAGTTCAAGTCCACCCTG-3’ |
|  | R: 5’-GCTTGATGTGGTTGCTCTCA -3’ |
| ACTN4#1 | F: 5’-GCCTGTAAGCCCAGCACTTTG-3’ |
|  | R: 5’-CCTCAGCCTCCCGAGTAGC-3’ |
| ACTN4#2 | F: 5’-GTTTGAGACCAGCCTGACCAAC-3’ |
|  | R: 5’-CTCCGCCTCCCAGGTTCAAG-3’ |
| ACTN4#3 | F: 5’-GTTTGAGACCAGCCTGACCAAC-3’ |
|  | R: 5’-CCTCAGCCTCCCGAGTAGC-3’ |
| FUSE#1 | F: 5’-GGGACCAAGGATGAGAAGAATG-3’ |
|  | R: 5’-TTGTTTGCTCCCTGAAATGATC-3’ |
| FUSE#2 | F: 5’-CACAAAATAAAAAATCCCGAGGGAATATA-3’ |
|  | R: 5’-CACATGATTTGTTTGCTCCCTG-3’ |
| FUSE#3 | F: 5’-AGGTGGTGGAGGGAGAG-3’ |
|  | R: 5’-TATATTCCCTCGGGATTTTTTATTTTGTG-3’ |
| GAPDH  promoter | F: 5’-TACTAGCGGTTTTACGGGCG-3’ |
|  | R: 5’-TCGAACAGGAGGAGCAGAGAGCGA-3’ |

**Table S2. Sequences of siRNAs and shRNAs used in this study.**

| **Definition** | **sequences** |
| --- | --- |
| si-circ#1 | ATGCAGAGGACCTTCACGG |
| si-circ#2 | CAGAGGACCTTCACGGCAT |
| si-NC | TTCTCCGAACGTGTCACGT |
| sh-USF2 | TCCAGACTGTAACGCAGACAA |
| sh-FUBP1 | GCTGCTTATTACGCTCACTAT |
| sh-FIR | CGTCCCAAGATGCTGTGTCTT |
| sh-ACTN4 | GCACCAACCTGAACAATGCCTTCGAA |

**Table S3. Sequences of pbobes used in this study.**

| **Method** | **sequences** | |
| --- | --- | --- |
| FISH | 5’Cy3-GCCGTGAAGGTCCTCTGCATC -3’Cy3 | |
| ISH | 5’Dig-GCCGTGAAGGTCCTCTGCATC-3’Dig | |
| Pull-down | olige | 5’-UUCUCCGAACGUGUCACGUTT- 3’ |
|  | CircACTN4 | 5’-AGGAGCAGCAUGAGCUUGAG - 3’ |
