# Supplementary figures and images for: The circACTN4 interacts with FUBP1 to promote tumorigenesis and progression of breast cancer by regulating the expression of proto-oncogene MYC
Source: Mol Cancer. 2021 Jun 11;20:91. doi: 10.1186/s12943-021-01383-x (PMC8194204; doi:10.1186/s12943-021-01383-x)

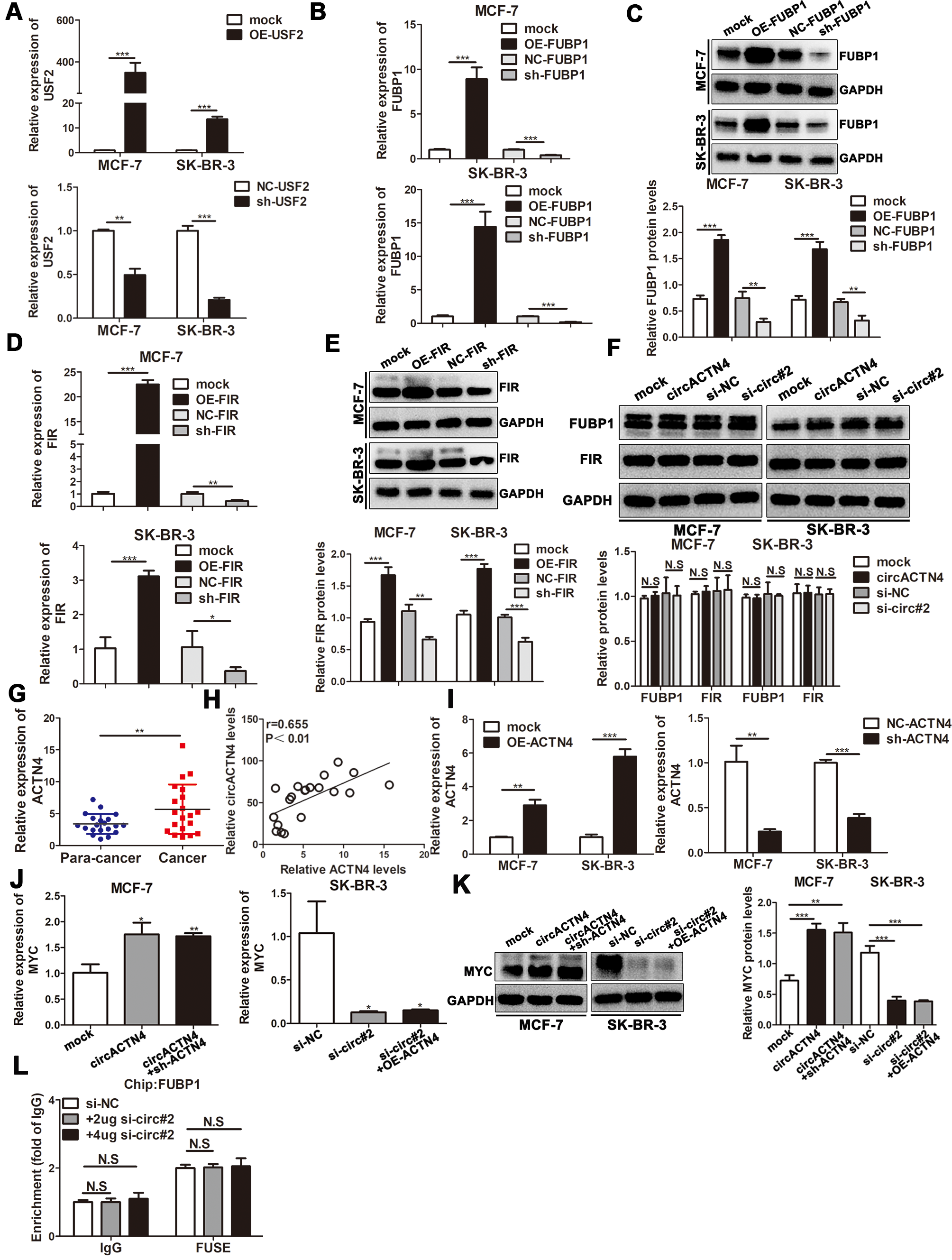

Supplement: Supplementary file 2 — Additional file 2: Figure S1. The expression levels of USF2, FUBP1, FIR, ACTN4 and MYC were determined by qRT-PCR or western blot and the binding of FUBP1 and FUSE was detected by CHIP-qPCR. a Relative expression of USF2 in BC cells was evaluated by qRT-PCR after overexpression and knockdown of USF2. b and c The expression of FUBP1 in BC cells was detected by qRT-PCR or western blot after overexpression and knockdown of FUBP1. d and e The expression level of FIR was determined in BC cells after overexpression and knockdown of FIR by qRT-PCR or western blot. f The expression levels of FUBP1 and FIR were detected in BC cells transfected with circACTN4 overexpression plasmids and si-circ#2 by western blot. g Relative expression of ACTN4 was determined in 20 pairs BC tissues and para-cancer tissues by qRT-PCR. h Pearson correlation analysis showed that the expression of ACTN4 was positively correlated with the level of circACTN4 in breast cancer tissues. i Relative expression of ACTN4 was evaluated in BC cells after transfection with ACTN4 overexpression or knockdown plasmids by qRT-PCR. j and k The expression level of MYC was detected after transfection or co-transfection with the indicated vectors and siRNA by qRT-PCR and western blot. l Chip assay showed that the transfection of BC cells with different concentrations of si-circ#2 had no effect on the binding of FUBP1 and FUSE. GAPDH was used as the normalizing gene in the above experiments. The data are presented as the mean ± SD, *P < 0.05, **P < 0.01, ***P < 0.001. [file 12943_2021_1383_MOESM2_ESM.tif]
